# Comparison of different models for estimation of direct and maternal genetic parameters on body weights in Awassi sheep

**DOI:** 10.5194/aab-65-121-2022

**Published:** 2022-03-17

**Authors:** Hatice Hızlı, Çiğdem Takma, Ertan Yazgan

**Affiliations:** 1 Department of Animal Breeding, Eastern Mediterranean Agricultural Research Institute, Adana, Turkey; 2 Department of Animal Science, Faculty of Agriculture, Ege University, Izmir, Turkey

## Abstract

The present study was conducted to estimate the (co)variance components for birth and weaning weight (BW and WW) in 8142 Awassi sheep between 2015 and 2017. Estimates were calculated with single-trait analysis by the average information restricted maximum likelihood (AI-REML) method, using a derivative-free algorithm by fitting six different univariate animal models. The negative of the log-likelihood function (LogL), Akaike information criterion (AIC), and Bayesian information criterion (BIC) tests were used for selecting the best fitted model. In addition, the goodness of fit between the two models was compared with the likelihood ratio test (LRT). Depending on the models, $h_{a}^{2}$ and $h_{m}^{2}$ ranged from 0.230 to 0.240 and 0.015 to 0.033 for BW, and 0.108 to 0.168 and 0.024 to 0.081 for WW, respectively. Model 3 for BW and Model 2 for WW were chosen as the best models by LogL comparison criteria. According to the LRT ratio test Model 2, Model 3, and Model 4 for BW and Model 2, Model 3, Model 4, Model 5, and Model 6 for WW were significant ( p<0.05 ). Including maternal genetic or maternal permanent environmental effects in these models was found to be significant in terms of parameter estimates.

## 1 Introduction

For maximizing the expected yield in livestock, the first condition is to apply selection programs in which the genetic parameters must be accurately estimated (Falconer and Mackay, 1996). Awassi sheep is one of the most common breeds grown in Turkey (TUIK, 2021). In addition, it is recognized as an important genetic resource for the sheep industry in more than 30 countries in the Middle East and all over the world (Galal et al., 2008). Consumers in the Middle East prefer Awassi mutton due to its taste. Falconer and Mackay (1996) have reported that many nongenetic factors play an important role in different quantitative characteristics. Birth weight (BW) and weaning weight (WW), which are quantitative characters, are important selection criteria for prenatal and postnatal growth performance in livestock breeding. In sheep breeding, the first 3-month weights during weaning of sheep are important production characteristics (Saatçi et al., 1999). Many researchers reported significant effects of lambing year, sex, and maternal age on BW and WW (Jawasreh and Khasawneh, 2007; Jawasreh et al., 2009; Aktaş and Doğan, 2014).

The yields to be improved by selection are affected by the genetic performance of the individual and environmental factors. In addition to reducing the effects of environmental factors to increase the degree of accuracy in selection, accurate estimation of the components that make up the genetic structure is required. In farm animals with long maternal lifespans, such as sheep, the phenotype of the offspring is determined not only by their genetic potential and environment, but also by the environment provided by their dams. The maternal effect on the offspring has been known since the beginning of animal breeding, and therefore it has been studied increasingly with developed software programs in recent years.

Direct additive, maternal additive, and maternal permanent environmental variances are considered important factors that determine the lamb's genetic gain and therefore performance (Willham, 1972; Meyer, 1992; Meyer and Kirkpatrick, 2005). In this connection, several studies have been done on heritability estimates and its constituent elements (Miraei-Ashtiani et al., 2007; Jafaroghli et al., 2010; Mohammadi et al., 2013a, b; Supakorn et al., 2013).

The aims of this study were to estimate the variance and covariance components, to compare different models for the BW–WW measurements of the Awassi lambs, and to reveal the importance of including maternal effects to design true breeding programs for genotypic selection of Awassi lambs. For this purpose, direct additive, maternal genetic and the maternal permanent environmental effects were calculated by applying six animal models.

## 2 Material and methods

### 2.1 Location and flock management

The study was carried out in Osmaniye province, districts, and villages between $36{}^{\circ }57^{\prime }$ – $37{}^{\circ }45^{\prime }$ N, $35{}^{\circ }52^{\prime }$ – $36{}^{\circ }42^{\prime }$ E, in a wide area covering an altitude of 2285 to 2400 m. Although the climate in the region differs in mountainous and lowland areas, it has the characteristic of the Mediterranean climate. In general, summers are hot and dry, and winters are mild and rainy. Average temperature is 18.2 ${}^{\circ }C$ , and average maximum temperature is 42.8 ${}^{\circ }C$ . Rainfall is higher in the winter and autumn months compared to other months, and the annual average rainfall is 767.6 mm.

Awassi is warm and very well adopted to arid conditions; it is one of Turkey's indigenous breeds with a high ability to adapt to different environments. Awassi sheep are raised in southeastern Anatolia, and the high milk production is one of the remarkable traits of the breed. Newborn lambs were kept together with their dams for three months (90 d) until weaning age. The birth weight (BW) and weaning weight (WW) at 3 months of age were recorded as growth traits. BW was taken within 24 h of the birth of lamb, and WW was taken on 3-month weights with digital hand weighing scales (up to 10 g sensitivity). The regional breeders kept their lambs in similar feeding and management conditions. Except for the winter period, they apply a feeding with a nomadic system based on the pasture and stubble grazing. They feed their animals mainly with hay and a small amount of grain in winter.

The material of this study was obtained from the Awassi Sub-Project in which elite herds were created in Osmaniye province within the scope of the national community-based animal improvement project. The records collected from 249 rams, 2416 sheep, and 8142 lambs between 2015–2017 years were used.

### 2.2 Statistical analysis for growth traits

A preliminary least-squares analysis of variance was performed with a general linear model (GLM) with SPSS software (IBM, 2020) to determine the main effects that will take place in the individual animal model. The least-squares analysis showed that the effects of sex of lambs (male, female), type of birth (single, twin), year of birth (2015, 2016, 2017), and age of dam (2, 3, 4, 5, 6, 7) were found to be statistically significant ( p<0.05 ) for BW and WW, and therefore these effects were included in the animal models. In these models sex of lambs, year of birth, and type of birth were considered as fixed effects and age of dam was used as a covariate for the estimations of BW. Also, sex of lambs, year of birth, type of birth, and age of dam were considered as fixed effects and BW of lamb was used as a covariate for the estimations of WW. The farm was not taken into account as a fixed effect in the models because the lambs that are the subject of this research were reared under similar care and feeding conditions and also the farms were located close to each other.

The estimates of variance components and genetic parameters for each trait were obtained from single-trait analysis with the average information restricted maximum likelihood (AI-REML) method, using a derivative-free algorithm by WOMBAT software (Meyer, 2012). The matrix representation of six single-trait animal models that used for genetic analysis were given as follows:

Model 1Y=Xβ+Zaα+εModel 2Y=Xβ+Zaα+Zmm+ε, with Cov(a,m)=0Model 3Y=Xβ+Zaα+Zmm+ε, with Cov(a,m)=AσamModel 4Y=Xβ+Zaα+Zcc+εModel 5Y=Xβ+Zaα+Zmm+Zcc+ε, with Cov(a,m)=0Model 6Y=Xβ+Zaα+Zmm+Zcc+ε, with Cov(a,m)=Aσam,

where Y is the vector of observations or records; β is the vector containing fixed effects such as sex, type of birth, year of birth, and age of dam; a , m , c , and ε are the vectors of direct additive genetic effects, maternal genetic effects, maternal permanent environmental effect of sheep and the residual, respectively. X , $Z_{a}$ , $Z_{m}$ , and $Z_{c}$ are the incidence matrices relating observations to b , a , m , and c , respectively. A is the numerator relationship matrix, and $\sigma_{\text{am}}$ is the covariance between direct additive and maternal genetic effects. The (co)variance structure of the random effects in the analysis can be described as

$$\begin{array}{c} V(a)=A\sigma_{a}^{2};\;V(m)=A\sigma_{m}^{2};\;V(c)=I_{d}\sigma_{c}^{2};\\ V(e)=I_{n}\sigma_{e}^{2};\;\text{Cov}(a,m)=A\sigma_{\text{am}}. \end{array}$$

Assumptions for (co)variance matrix involving random effects can be described as A is the numerator relationship matrix, $\sigma_{a}^{2}$ is the direct additive genetic variance, $\sigma_{m}^{2}$ is the maternal additive genetic variance, $\sigma_{\text{am}}$ is the direct-maternal additive genetic covariance, $\sigma_{c}^{2}$ is the maternal permanent environmental variance, $\sigma_{e}^{2}$ is the residual variance, and $I_{d}$ and $I_{n}$ are the identity matrices of an order equal to the number of dams and records, respectively.

The total heritability ( $h_{T}^{2}$ ) for each model was calculated as $h_{T}^{2}=(\sigma_{a}^{2}+0.5\sigma_{m}^{2}+1.5\sigma_{\text{am}})/\sigma_{p}^{2}$ , which was defined by Willham (1972). The genetic correlation between the direct additive genetic and the maternal additive genetic effects ( $r_{\text{am}}$ ) was estimated as the ratio of the estimates of the $\sigma_{\text{am}}$ to the estimates of $\sigma_{a}^{2}$ and $\sigma_{m}^{2}$ (Falconer and Mackay, 1996).

The starting values of variance parameters for REML iterations were obtained with ANOVA estimators (Searle et al., 2006). The most appropriate model for convergence of REML solutions was assumed when the average difference in likelihood functions in successive iterations was less than $10^{-8}$ . The analysis was continued by taking the previous starting values until ensuring the estimates did not change and confirming the convergence was met (Meyer, 1991, 2007).

The log-likelihood (LogL) test, Akaike information criterion (AIC), and Bayesian information criterion (BIC) were performed to determine the best model. These likelihood-based tests are AIC=-2⋅LogL+2⋅p and BIC=-2⋅LogL+p⋅log⁡(N-r(x)) , and where p is the number of predicted parameters, N is the sample size, and r(x) is the rank of the coefficient matrix for the fixed effects in the model (Akaike, 1973; Maniatis et al., 2013; Meyer, 1992; López-Romero and Carabano, 2003; Schwarz, 1978). The model with the highest LogL or minimum AIC and BIC value is considered the best model (Pham, 2019). Moreover, the likelihood ratio test (LRT) is a statistical test of the goodness of fit between two models; a relatively full model is compared to a reduced model to determine importance of a parameter that gives rise to increase in log-likelihood. That is, the full model must differ from the reduced model only by the addition of one or more parameters. In other words, LRT explains whether it is good to add a parameter to a model or not. The test begins with a comparison of the likelihood scores of the two models, after which follows a chi-squared distribution ( $\chi^{2}$ ) with degrees of freedom equal to the difference in dimensionality of the models (Felsenstein, 1981; Huelsenbeck and Crandall, 1997). The equation for the test statistic is as follows:

$$\text{LR}=-2\cdot {\text{Loglikelihood}}_{\text{(reduced)}}-{\text{Loglikelihood}}_{\text{(full)}}.$$

In this study, LogL differences between Model 1 versus other models were examined with LRT in order to determine the importance of the maternal additive genetic and maternal permanent environmental effects.

## 3 Results and discussion

The structure of the data set used in this study is presented in Table 1. According to descriptive statistics the coefficient of variation for WW is much lower than BW (Table 1).

**Table 1:** The data structure of BW and WW for Awassi lambs.

	Traits	
Item	BW	WW
Mean	4.15	24.42
SD	0.69	2.65
SE	0.007	0.029
CV	16.5	10.83
Min	2.24	17.42
Max	6.06	31.3
No. of records	8142	7647
No. of valid records	8142	7647
No. of lamb	8142	7647
No. of ram	249	243
No. of sheep	2416	2360

SD: standard deviation, SE: standard error, CV: coefficient of variance, Min: minimum and Max: maximum.

Least-squares means for fixed effects and standard errors for BW and WW are shown in Table 2, and they were estimated for BW and WW as 3.87±0.01 and 24.49±0.57 , respectively. Sex of lambs had a significant effect on BW and WW ( p<0.01 ). Vatankhah and Demand (2008a, b), Yavarifard et al. (2015), and Jalil-Sarghale et al. (2014) reported similar results. The result of variance analysis showed that the year of birth had significant effects on BW ( p<0.01 ), but not significant on WW ( p>0.05 ). Also, age of dam had a significant effect on BW and WW ( p<0.001 ). Single-born lambs had higher body weights than twins, as expected. Similar results for age of dams and sex of offspring's growth in different species have been reported by many researchers in the literature (Gholizadeh et al., 2010; Jawasreh et al., 2018; Rashidi et al., 2011; Mohammadi et al., 2013c; Mohammadabadi and Sattayimokhtari, 2013).

**Table 2:** The least-squares means (LSMs) and standard errors (SEs) of sex, birth type, year, and age of dam effects for Awassi lambs' BW and WW.

	BW/kg		WW/kg	
Fix effects	N	LSM ± SE	N	LSM ± SE
	8142	3.87±0.01	7647	24.49±0.57
Sex				
Female	3980	4.07±0.01	3885	24.17±0.04
Male	4162	4.23±0.01	4062	24.65±0.04
p		<0.001		<0.001
Birth type				
Single	7534	4.20±0.01	7354	24.40±0.03
Twin	608	3.55±0.03	593	24.61±0.12
p		<0.001		<0.001
Year				
2015	2069	$4.11\pm 0.01^{b}$	2020	$24.33\pm 0.06^{b}$
2016	3088	$4.15\pm 0.01^{a}$	3014	$24.33\pm 0.05^{b}$
2017	2985	$4.18\pm 0.01^{a}$	2913	$24.57\pm 0.05^{a}$
p		<0.001		<0.05
Age of dam				
b	b (age of dam)	-0.041±0.005	b (BW)	$1.20\pm 0.04^{a}$
2			1388	$24.44\pm 0.07^{b}$
3			1609	$24.47\pm 0.06^{b}$
4			1989	$24.71\pm 0.06^{a}$
5			1231	$24.18\pm 0.07^{c}$
6			1058	$24.00\pm 0.08^{c}$
7			672	$24.51\pm 0.1^{\text{ab}}$
p		<0.001		<0.001
$R^{2}$	0.09		0.11	

${}^{a,\;b,\;c}$ Different letters in the same column indicate that the difference is significant ( p<0.05 ).

**Table 3:** Estimates of (co)variance components and genetic parameters for BW of Awassi lambs obtained in different models (mean ± standard error). The best models according to LogL, AIC and BIC criteria are shown in italic.

Trait	Models					
BW	Model 1	Model 2	Model 3	Model 4	Model 5	Model 6
$\sigma_{a}^{2}$	0.12	0.117	0.115	0.117	0.117	0.116
$\sigma_{e}^{2}$	0.38	0.368	0.37	0.368	0.369	0.385
$\sigma_{p}^{2}$	0.5	0.5	0.5	0.5	0.5	0.49
$\sigma_{m}^{2}$		0.015	0.015		0.0073	0.016
$\sigma_{c}^{2}$				0.0145	0.0073	0.001
$\sigma_{\text{am}}$			-0.013			-0.029
$h_{a}^{2}$	0.240±0.03	0.234±0.03	0.230±0.03	0.234±0.027	0.234±0.027	0.237±0.028
$h_{m}^{2}$		0.030±0.014	0.030±0.014		0.015±0.001	0.033±0.150
$h_{c}^{2}$				0.030±0.014	0.015±0.014	0.002±0.151
$h_{T}^{2}$	0.240	0.249	0.206	0.234	0.241	0.164
$C_{\text{am}}$			-0.026			-0.059
$r_{\text{am}}$			-0.299			-0.294
LogL	-1175.34	-1172.38	-1171.73	-1173.02	-1173.02	-1171.79
AIC	2354.68	*2350.76*	2351.46	2352.04	2354.04	2353.58
BIC	*2368.68*	2371.76	2379.48	2373.06	2382.06	2388.6
$\chi_{A1-A2,\ldots A6}^{2}$	–	5.92 ${}^{*}$	7.22 ${}^{*}$	4.64 ${}^{*}$	4.64	7.10

$\sigma_{a}^{2}$ : direct additive genetic variance; $\sigma_{m}^{2}$ : maternal additive genetic variance; $\sigma_{\text{am}}$ : direct–maternal genetic covariance; $\sigma_{c}^{2}$ : maternal permanent environmental variance; $\sigma_{e}^{2}$ : error variance; $\sigma_{p}^{2}$ : phenotypic variance; $h_{a}^{2}$ : direct heritability; $h_{m}^{2}$ : maternal heritability; $C_{\text{am}}$ : $\sigma_{\text{am}}/\sigma_{p}^{2}$ ; $r_{\text{am}}$ : genetic correlation between direct and maternal effects; $h_{c}^{2}$ : $\sigma_{c}^{2}/\sigma_{p}^{2}$ maternal permanent environmental variance as proportion of phenotypic variance; $h_{T}^{2}$ : total heritability: $(\sigma_{a}^{2}+0.5\sigma_{m}^{2}+1.5\sigma_{\text{am}})/\sigma_{p}^{2}$ , $\chi^{2}=-2\cdot {\text{Loglikelihood}}_{\text{(reduced)}}-{\text{Loglikelihood}}_{\text{(full)}}$ ; ${}^{*}$ : test value is significant at the 0.05 level.

Estimates of (co)variance components and genetic parameters for BWs of Awassi lambs obtained with different models are presented in Table 3. The direct additive genetic variance was found to be lower in Models 2, 3, 4, 5, and 6 when compared with Model 1. Similar results have been reported for various goat and sheep breeds (Saatçi et al., 1999; Tesema et al., 2020; Szwaczkowski et al., 2006). Estimates of maternal permanent environmental variances in Models 4, 5, and 6 for BW were as 0.0145, 0.0073, and 0.001, respectively. The estimates of $C_{\text{am}}$ , which is the ratio of covariance between additive genetic and maternal genetic variances in phenotypic variance for Models 3 and 6, were estimated as -0.026 and -0.059 , respectively.

Correlations between direct additive genetic and maternal genetic effects were calculated as -0.299 and -0.294 for BW in Model 3 and 6, respectively. These similar negative estimates have been reported in many studies (Ligda et al., 2000; Zhang et al., 2009; Gowane et al., 2010; Tesema et al., 2020). Meyer (1992) and Heydarpour et al. (2008) reported that higher and negative predictions of correlations are due to the presence of true genetic antagonism between components, poor data structure with few progenies per dam, and limited information about the performance of linked offspring and dams. Therefore, both additive genetic and maternal genetic effects must be considered in selection programs to maximize genetic gain (Meyer, 1992). Gutierrez et al. (1997) also suggested that this relationship should be compensated by improving management practices and using supplementary feeding.

In addition, the most suitable model was selected in italics as the best model according to LogL, AIC, and BIC criteria for the estimation of genetic parameters for the BW and WW of Awassi lambs (Tables 3 and 4). Models 1, 2, and 3 were mentioned as the best model according to BIC, AIC, and LogL criteria, respectively. Also, Models 2, 3, and 4 were found to be more statistically significant than Model 1 according to LRT ( p<0.05 ). The attained results point out that maternal genetic effects or maternal permanent environmental effects should be included into the models for genetic evaluation of Awassi BW.

**Table 4:** Estimates of (co)variance components and genetic parameters for WW of Awassi lambs obtained in different models (mean ± standard error). The best models according to LogL, AIC and BIC criteria are shown in italic.

Trait	Models					
WW	Model 1	Model 2	Model 3	Model 4	Model 5	Model 6
$\sigma_{a}^{2}$	440.08	439.73	438.37	437.80	437.29	285.79
$\sigma_{e}^{2}$	2175.20	2066.10	2045.30	2052.20	2052.60	2212.20
$\sigma_{p}^{2}$	2615.30	2614.80	2630.00	2615.70	2615.60	2644.00
$\sigma_{m}^{2}$		108.04	122.23		62.836	0.095
$\sigma_{c}^{2}$				125.67	62.836	146.055
$\sigma_{\text{am}}$			24.10			-0.03
$h_{a}^{2}$	0.168±0.029	0.168±0.029	0.167±0.029	0.167±0.029	0.167±0.029	0.108±0.023
$h_{m}^{2}$		0.041±0.013	0.050±0.014		0.024±0.0001	0.081±0.183
$h_{c}^{2}$				0.048±0.015	0.024±0.015	0.055±0.015
$h_{T}^{2}$	0.168	0.168	0.204	0.167	0.179	0.108
$C_{\text{am}}$			0.009			0.000
$r_{\text{am}}$			0.018			-0.062
LogL	-36029.91	-36024.10	-36024.21	-36024.81	-36024.81	-36035.86
AIC	72 063.82	*72 054.2*	72 056.42	72 055.62	72 057.62	72 081.72
BIC	72 077.82	*72 075.2*	72 084.42	72 076.64	72 085.64	72 116.74
$\chi_{A1-A2,\ldots A6}^{2}$	–	11.62 ${}^{*}$	11.4 ${}^{*}$	10.2 ${}^{*}$	10.2 ${}^{*}$	11.9 ${}^{*}$

$\sigma_{a}^{2}$ : direct additive genetic variance; $\sigma_{m}^{2}$ : maternal additive genetic variance; $\sigma_{\text{am}}$ : direct–maternal genetic covariance; $\sigma_{c}^{2}$ : maternal permanent environmental variance; $\sigma_{e}^{2}$ : error variance; $\sigma_{p}^{2}$ : phenotypic variance; $h_{a}^{2}$ : direct heritability; $h_{m}^{2}$ : maternal heritability; $C_{\text{am}}$ : $\sigma_{\text{am}}/\sigma_{p}^{2}$ ; $r_{\text{am}}$ : genetic correlation between direct and maternal effects; $h_{c}^{2}$ : $\sigma_{c}^{2}/\sigma_{p}^{2}$ maternal permanent environmental variance as proportion of phenotypic variance; $h_{T}^{2}$ : total heritability: $(\sigma_{a}^{2}+0.5\sigma_{m}^{2}+1.5\sigma_{\text{am}})/\sigma_{p}^{2}$ , $\chi^{2}=-2\cdot {\text{Loglikelihood}}_{\text{(reduced)}}-{\text{Loglikelihood}}_{\text{(full)}}$ ${}^{*}$ : test value is significant at the 0.05 level.

In Table 4 the results showed that Model 2 was selected as the best model with respect to all criteria. Also, Models 2, 3, 4, 5, and 6 were found to be significant compared to Model 1 according to LRT ( p<0.05 ). Moreover, estimates of (co)variance components and genetic parameters for WW of Awassi lambs obtained in different models are presented in Table 4. Table 4 shows that Models 5 and 6, in which maternal additive genetic and maternal permanent environmental variances are added, have lower $\sigma_{a}^{2}$ and $h_{a}^{2}$ values than Model 1 for WW. The estimates of $\sigma_{a}^{2}$ and $h_{a}^{2}$ in Models 2 and 3, which have maternal additive genetic variance and not maternal permanent environmental variance, were found as 439.73±0.029 , 0.168±0.029 , and 438.37±0.029 , 0.167±0.029 , respectively. In Models 2, 3, 4, 5, and 6, the maternal genetic variance and the maternal permanent environmental variance estimates of $\sigma_{a}^{2}$ and $h_{a}^{2}$ were found to be smaller than Model 1. $C_{\text{am}}$ estimates for Models 3 and 6 were estimated to be 0.009 and almost zero, respectively. Similar results have been reported by Jawasreh et al. (2018b).

In Model 4, 5, and 6 the $h_{c}^{2}$ estimates were found as 0.048±0.015 , 0.024±0.015 , and 0.055±0.015 , respectively. Correlations between additive genetic and maternal genetic effects were obtained as 0.018 and -0.062 in Model 3 and 6 for WW, respectively.

The $h_{T}^{2}$ estimates were 0.168±0.015 , 0.167±0.015 , 0.204±0.015 , 0.167±0.015 , 0.179±0.015 , and 0.108±0.015 in Models 1, 2, 3, 4, 5, and 6, respectively. The $h_{T}^{2}$ estimate was the highest in Model 3 and the lowest in Model 6. Prakash et al. (2016) reported that the maternal genetic variances are significant but are not generally considered. Therefore, the heritability estimates are biased upward and the resulting selection efficiency is reduced, but division of the (co)variances is made more accurate as it enables the estimation of the contribution of each individual effect to the overall performance of the animal.

In present study, $h_{a}^{2}$ values were estimated from 0.230 to 0.240, $h_{m}^{2}$ from 0.015 to 0.033, $h_{c}^{2}$ from 0.002 to 0.030, $r_{\text{am}}$ from -0.294 to -0.299 , and $h_{T}^{2}$ from 0.164 to 0.240 for BW in Table 3. Similarly, $h_{a}^{2}$ , $h_{m}^{2}$ , $h_{c}^{2}$ , $r_{\text{am}}$ , and $h_{T}^{2}$ were estimated with values ranging from 0.108 to 0.168, 0.024–0.081, 0.024–0.055, -0.062 –0.018, and 0.108–0.168, respectively for WW (Table 4). Şireli et al. (2015) found $h_{a}^{2}$ and $h_{m}^{2}$ for BW between 0.21–0.57 and 0.09–0.18, respectively, and for WW were between 0.02–0.13 and 0.03–0.17, respectively. Jawasreh et al. (2018) estimated $h_{T}^{2}$ for BW and WW as 0.30 and 0.19, respectively, and $\sigma_{m}^{2}$ values for BW and WW were found as 0.13 and 0.03, respectively, in their study. While our estimates of (co)variance components and genetic parameters for BW and WW of Awassi lambs obtained by different models were similar with the findings of Şireli et al. (2015), it was smaller than the findings of Jawasreh et al. (2018).

Tables 3 and 4 show that the estimates of maternal genetic and maternal permanent environmental effects for WW were increased when compared with the estimates for BW. Also, Tables 3 and 4 show that the $r_{\text{am}}$ , $h_{a}^{2}$ , and $h_{T}^{2}$ estimates of WW were lower than the estimates for BW. The lower variance component estimates explain the magnitude $h_{m}^{2}$ and $h_{c}^{2}$ estimates for WW. While $h_{m}^{2}$ can be transferred from generation to generation, $h_{c}^{2}$ is determined by factors that cannot be transferred from generation to generation. The maternal environmental effect is the factors that are not transmitted from generation to generation and only affect the offspring, as maternal nutrition, uterine volume, litter size, and maternal care (Çelikeloğlu and Tekerli, 2014). Hanrahan (1976) and Meyer (1992) also reported that maternal effects explain most of the variation in their early age studies, which is similar to the results of this study.

## 4 Conclusions

This research was conducted to compare different models based on predictions of genetic parameters and to show whether maternal effects could better explain the genetic effects on BW and WW traits. Overestimation of phenotypic variation, in other words, low total heritability, may be due to the low litter sizes, inconsistency in records, and poor maintenance, feeding, and herd management in the farms. It is thought that by improving these factors, the reliability in estimations would be increased.

Consequently, the maternal effect should be taken into consideration for the purpose of improving accuracy in parameter estimations and therefore increasing the success of breeding programs.

## Supplement

10.5194/aab-65-121-2022-supplement The supplement related to this article is available online at: https://doi.org/10.5194/aab-65-121-2022-supplement.

## Data Availability

The data can be requested with written permission from the Republic of Turkey, General Directorate of Agricultural Research and Policies of the Ministry of Food, Agriculture and Livestock (, last access: 14 March 2021).

## Code availability

SPSS software (version 20.0) is available at: , last access: 14 March 2021 (IBM, 2020) and WOMBAT at: , last access: 14 March 2021.
