# Supplementary material for: Comparison of different models for estimation of direct and maternal genetic parameters on body weights in Awassi sheep
Source: Arch Anim Breed. 2022 Mar 17;65(1):121–8. doi: 10.5194/aab-65-121-2022 (PMC8949804; doi:10.5194/aab-65-121-2022)
Supplement: The supplement related to this article is available online at: https://doi.org/10.5194/aab-65-121-2022-supplement. [file aab-65-121-supplement.pdf]

Supplement of Arch. Anim. Breed., 65, 121–128, 2022
https://doi.org/10.5194/aab-65-121-2022-supplement
© Author(s) 2022. CC BY 4.0 License.

Open Access
Archives Animal Breeding

*Supplement of*

# Comparison of different models for estimation of direct and maternal genetic parameters on body weights in Awassi sheep

**Hatice Hızlı et al.**

*Correspondence to:* Hatice Hızlı (hatice.hizlibostan@tarimorman.gov.tr)

The copyright of individual parts of the supplement might differ from the article licence.

Tablo 1. The data structure of BW, WW for Awassi lambs

| | Traits | |
|---|---|---|
| Item | BW | WW |
| Mean | 4.15 | 24.42 |
| SD | 0.69 | 2.65 |
| SE | 0.007 | 0.029 |
| CV | 16.5 | 10.83 |
| Min | 2.24 | 17.42 |
| Max | 6.06 | 31.3 |
| no of records | 8142 | 7647 |
| No of valid records | 8142 | 7647 |
| No of lamb | 8142 | 7647 |
| No of ram | 249 | 243 |
| No of sheep | 2416 | 2360 |

SD: standard deviation, SE: Standart Error, CV: Coefficient of variance, Min: minimum and Max: Maximum

Tablo 2. The least-squares means (LSM) and standard errors (SE) of gender, birth type, year, and age of dam effects for Awassi lambs’ BW and WW

| | BW/kg | | WW/kg | |
|---|---|---|---|---|
| Fix effects | N | LSM ± SE | N | LSM ± SE |
| | 8142 | 3.87 ± 0.01 | 7647 | 24.49 ± 0.57 |
| Gender | | | | |
| Female | 3980 | 4.07 ± 0.01 | 3885 | 24.17 ± 0.04 |
| Male | 4162 | 4.23 ± 0.01 | 4062 | 24.65 ± 0.04 |
| p | | < 0.001 | | < 0.001 |
| Birth type | | | | |
| Single | 7534 | 4.20 ± 0.01 | 7354 | 24.40 ± 0.03 |
| Twin | 608 | 3.55 ± 0.03 | 593 | 24.61 ± 0.12 |
| p | | < 0.001 | | <0.001 |
| Year | | | | |
| 2015 | 2069 | 4.11 ± 0.01$^{b}$ | 2020 | 24.33 ± 0.06$^{b}$ |
| 2016 | 3088 | 4.15 ± 0.01$^{a}$ | 3014 | 24.33 ± 0.05$^{b}$ |
| 2017 | 2985 | 4.18 ± 0.01$^{a}$ | 2913 | 24.57 ± 0.05$^{a}$ |
| p | | < 0.001 | | < 0.05 |
| Age of dam | | | | |
| b | b (Age of dam) | -0.041±0.005 | b (BW) | 1.20 ± 0.04$^{a}$ |
| 2 | | | 1388 | 24.44 ± 0.07$^{b}$ |
| 3 | | | 1609 | 24.47 ± 0.06$^{b}$ |
| 4 | | | 1989 | 24.71 ± 0.06$^{a}$ |
| 5 | | | 1231 | 24.18 ± 0.07$^{c}$ |
| 6 | | | 1058 | 24.00 ± 0.08$^{c}$ |
| 7 | | | 672 | 24.51 ± 0.1$^{ab}$ |
| p | | <0.001 | | <0.001 |
| R Squared | | 0.09 | | 0.11 |

Table 3. Estimates of (co)variance components and genetic parameters for BW of Awassi lambs obtained in different models (mean ± standard error)

| Trait | Models | | | | | |
|---|---|---|---|---|---|---|
| BW | Model 1 | Model 2 | Model 3 | Model 4 | Model 5 | Model 6 |
| $\sigma_a^2$ | 0.12 | 0.117 | 0.115 | 0.117 | 0.117 | 0.116 |
| $\sigma_e^2$ | 0.38 | 0.368 | 0.37 | 0.368 | 0.369 | 0.385 |
| $\sigma_p^2$ | 0.5 | 0.5 | 0.5 | 0.5 | 0.5 | 0.49 |
| $\sigma_m^2$ | | 0.015 | 0.015 | | 0.0073 | 0.016 |
| $\sigma_c^2$ | | | | 0.0145 | 0.0073 | 0.001 |
| $\sigma_{am}$ | | | -0.013 | | | -0.029 |
| $h_a^2$ | 0.240 ± 0.03 | 0.234 ± 0.03 | 0.230 ± 0.03 | 0.234 ± 0.027 | 0.234 ± 0.027 | 0.237 ± 0.028 |
| $h_m^2$ | | 0.030 ± 0.014 | 0.030 ± 0.014 | | 0.015 ± 0.001 | 0.033 ± 0.150 |
| $h_c^2$ | | | | 0.030 ± 0.014 | 0.015 ± 0.014 | 0.002 ± 0.151 |
| $h_T^2$ | 0.240 | 0.249 | 0.206 | 0.234 | 0.241 | 0.164 |
| $C_{am}$ | | | -0.026 | | | -0.059 |
| $r_{am}$ | | | -0.299 | | | -0.294 |
| LogL | -1175.34 | -1172.38 | **-1171.73** | -1173.02 | -1173.02 | -1171.79 |
| AIC | 2354.68 | **2350.76** | 2351.46 | 2352.04 | 2354.04 | 2353.58 |
| BIC | **2368.68** | 2371.76 | 2379.48 | 2373.06 | 2382.06 | 2388.6 |
| $\chi^2_{A1-A2,\ldots A6}$ | - | 5.92* | 7.22* | 4.64* | 4.64 | 7.10 |

$\sigma_a^2$: direct additive genetic variance; $\sigma_m^2$: maternal additive genetic variance; $\sigma_{am}$: direct-maternal genetic covariance; $\sigma_c^2$: maternal permanent environmental variance; $\sigma_e^2$: error variance; $\sigma_p^2$: phenotypic variance; $h_a^2$: direct heritability; $h_m^2$ : maternal heritability; $C_{am}$:$\sigma_{am}$ / $\sigma^2_p$; $r_{am}$ : genetic correlation between direct and maternal effects; $h_c^2$: $\sigma_c^2/\sigma_p^2$ maternal permanent environmental variance as proportion of phenotypic variance; $h_T^2$: Total heritability: $(\sigma_a^2 + 0.5\sigma_m^2 + 1.5\sigma_{am})/\sigma_p^2$, $\chi^2 = -2 * Loglikelihood_{(reduced)} - Loglikelihood_{(full)}$; *: Test value is significantly at the 0.05 level.

Table 4. Estimates of (co)variance components and genetic parameters for WW of Awassi lambs obtained in different models (mean ± standard error)

| Trait | Models | | | | | |
|---|---|---|---|---|---|---|
| WW | Model 1 | Model 2 | Model 3 | Model 4 | Model 5 | Model 6 |
| $\sigma_a^2$ | 440.08 | 439.73 | 438.37 | 437.80 | 437.29 | 285.79 |
| $\sigma_e^2$ | 2175.20 | 2066.10 | 2045.30 | 2052.20 | 2052.60 | 2212.20 |
| $\sigma_p^2$ | 2615.30 | 2614.80 | 2630.00 | 2615.70 | 2615.60 | 2644.00 |
| $\sigma_m^2$ | | 108.04 | 122.23 | | 62.836 | 0.095 |
| $\sigma_c^2$ | | | | 125.67 | 62.836 | 146.055 |
| $\sigma_{am}$ | | | 24.10 | | | -0.03 |
| $h_a^2$ | 0.168 ± 0.029 | 0.168 ± 0.029 | 0.167 ± 0.029 | 0.167 ± 0.029 | 0.167 ± 0.029 | 0.108 ± 0.023 |
| $h_m^2$ | | 0.041 ± 0.013 | 0.050 ± 0.014 | | 0.024 ± 0.0001 | 0.081 ± 0.183 |
| $h_c^2$ | | | | 0.048 ± 0.015 | 0.024 ± 0.015 | 0.055 ± 0.015 |
| $h_T^2$ | 0.168 | 0.168 | 0.204 | 0.167 | 0.179 | 0.108 |
| $C_{am}$ | | | 0.009 | | | 0.000 |
| $r_{am}$ | | | 0.018 | | | -0.062 |
| LogL | -36029.91 | **-36024.10** | -36024.21 | -36024.81 | -36024.81 | -36035.86 |
| AIC | 72063.82 | **72054.2** | 72056.42 | 72055.62 | 72057.62 | 72081.72 |
| BIC | 72077.82 | **72075.2** | 72084.42 | 72076.64 | 72085.64 | 72116.74 |
| $\chi^2_{A1-A2,\ldots A6}$ | - | 11.62* | 11.4* | 10.2* | 10.2* | 11.9* |

$\sigma_a^2$: direct additive genetic variance; $\sigma_m^2$: maternal additive genetic variance; $\sigma_{am}$: direct-maternal genetic covariance; $\sigma_c^2$: maternal permanent environmental variance; $\sigma_e^2$: error variance; $\sigma_p^2$: phenotypic variance; $h_a^2$: direct heritability; $h_m^2$ : maternal heritability; $C_{am}$:$\sigma_{am}$ / $\sigma^2_p$; $r_{am}$ : genetic correlation between direct and maternal effects; $h_c^2$: $\sigma_c^2/\sigma_p^2$ maternal permanent environmental variance as proportion of phenotypic variance; $h_T^2$: total heritability: $(\sigma_a^2 + 0.5\sigma_m^2 + 1.5\sigma_{am})/\sigma_p^2$, $\chi^2 = -2 * Loglikelihood_{(reduced)} - Loglikelihood_{(full)}$ *: Test value is significantly at the 0.05 level.
